# Supplementary material for: Transient Waterlogging Events Impair Shoot and Root Physiology and Reduce Grain Yield of Durum Wheat Cultivars
Source: Plants (Basel). 2021 Nov 1;10(11):2357. doi: 10.3390/plants10112357 (PMC8625979; doi:10.3390/plants10112357)
Supplement: Supplementary file 1 [file plants-10-02357-s001.zip › plants-1379428-supplementary.pdf]

plants

MDPI

Supplementary Material

Table S1. Principal growth stages of durum wheat cultivars Emilio Lepido and Svevo subjected to 0, 14 or 35 days of waterlogging (C, WL14, WL35).

Growth stage	BBCH code	Emilio Lepido			Svevo		
		C	WL14	WL35	C	WL14	WL35
Beginning of tillering	20	24 February	24 February	24 February	24 February	24 February	24 February
First node detectable	31	10 March	10 March	10 March	10 March	10 March	10 March
Flowering	65	6 May	6 May	15 May	6 May	6 May	15 May
Maturity	99	29 June	29 June	29 June	29 June	29 June	29 June

Table S2. Physiological parameters of the durum wheat cultivars Emilio Lepido and Svevo subjected to 0, 14 or 35 days of waterlogging (C, WL14, and WL35, respectively), measured during recovery (70 days from the beginning of waterlogging).

F values and *p* levels (***: $p \leq 0.001$, **: $p \leq 0.01$, *: $p \leq 0.05$, ns: $p > 0.05$) of two-way analysis of variance (ANOVA) for the effects of cultivar (*C*; degrees of freedom, df: 1), waterlogging (*WL*; df: 2) and their interaction (*C* × *WL*; df: 2) on parameters are shown. In case two-way ANOVA reveals a significant *C* × *WL* interactive effect on the specific parameter, according to Tukey's *post hoc* test, different letters indicate significant differences among means ($p \leq 0.05$).

Parameter	Emilio Lepido			Svevo			*ANOVA*		
	C	WL14	WL35	C	WL14	WL35	*C*	*WL*	*C* × *WL*
**A**	9.3±0.8 a	14.2±1.4 c	9.7±0.1 a	9.9±0.7 ab	12.5±0.2 bc	13.2±2.3 c	2.75 ns	19.83 ***	9.58 ***
**g_s**	0.13±0.02	0.18±0.02	0.18±0.04	0.16±0.03	0.20±0.00	0.24±0.03	13.50 **	14.29 ***	1.60 ns
**C_i**	262±2	251±5	285±19	275±23	278±1	289±6	7.88 *	6.88 **	1.73 ns
**WUE_{in}**	73±3	77±1	57±13	64±15	62±1	55±3	6.70 ns	6.10 **	1.30 ns
**F_v/F_m**	0.78±0.01	0.79±0.01	0.80±0.00	0.78±0.00	0.79±0.02	0.78±0.01	2.02 ns	0.77 ns	1.77 ns
**Φ_{PSII}**	0.56±0.01 a	0.56±0.03 a	0.64±0.01 c	0.59±0.01 ab	0.60±0.02 bc	0.62±0.03 bc	4.23 ns	21.19 ***	7.50 **
**qP**	0.80±0.01 ab	0.80±0.02 a	0.89±0.01 c	0.85±0.00 bc	0.85±0.01 bc	0.87±0.05 c	8.41 **	17.95 ***	6.91 **
**qNP**	0.46±0.00 b	0.46±0.06 b	0.34±0.02 a	0.46±0.01 b	0.35±0.04 a	0.29±0.03 a	13.39 **	34.38 ***	5.56 *

Parameters: A, CO_2 assimilation rate (μmol m^{-2} s^{-1}); g_s, stomatal conductance (mol m^{-2} s^{-1}); C_i, intercellular CO_2 carbon concentration (μmol mol^{-1}); WUE_{in}, intrinsic water use efficiency (i.e, A/g_s; μmol mol^{-1}); F_v/F_m, maximum quantum efficiency of the photosystem II (PSII) photochemistry; Φ_{PSII}, PSII operating efficiency in light conditions; qP, photochemical quenching; qNP, non-photochemical quenching.
